# Advancing Behavioral HIV Prevention: Adapting an Evidence-Based Intervention for People Living with HIV and Alcohol Use Disorders

**DOI:** 10.1155/2015/879052

**Published:** 2015-12-01

**Authors:** M. L. Armstrong, A. M. LaPlante, F. L. Altice, M. Copenhaver, P. E. Molina

**Affiliations:** ^1^Comprehensive Alcohol Research Center and Alcohol and Drug Abuse Center, Department of Physiology, LSUHSC, New Orleans, LA 70112, USA; ^2^Yale University School of Medicine, Section of Infectious Diseases, AIDS Program, New Haven, CT 06510, USA; ^3^Yale University School of Public Health, Division of Epidemiology of Microbial Diseases, New Haven, CT 06510, USA; ^4^Center for Health, Intervention, & Prevention (CHIP), University of Connecticut, Storrs, CT 06269, USA

## Abstract

Alcohol use disorders (AUDs) are highly prevalent among people living with HIV/AIDS (PLWHA) and are associated with increased HIV risk behaviors, suboptimal treatment adherence, and greater risk for disease progression. We used the ADAPT-ITT strategy to adapt an evidence-based intervention (EBI), the Holistic Health Recovery Program (HHRP+), that focuses on secondary HIV prevention and antiretroviral therapy (ART) adherence and apply it to PLWHA with problematic drinking. Focus groups (FGs) were conducted with PLWHA who consume alcohol and with treatment providers at the largest HIV primary care clinic in New Orleans, LA. Overall themes that emerged from the FGs included the following: (1) negative mood states contribute to heavy alcohol consumption in PLWHA; (2) high levels of psychosocial stress, paired with few adaptive coping strategies, perpetuate the use of harmful alcohol consumption in PLWHA; (3) local cultural norms are related to the permissiveness and pervasiveness of drinking and contribute to heavy alcohol use; (4) healthcare providers unanimously stated that outpatient options for AUD intervention are scarce, (5) misperceptions about the relationships between alcohol and HIV are common; (6) PLWHA are interested in learning about alcohol's impact on ART and HIV disease progression. These data were used to design the adapted EBI.

## 1. Introduction

Alcohol consumption, especially among individuals with alcohol use disorders (AUDs), is independently associated with sexual HIV risk and with HIV transmission [1, 2]. People living with HIV/AIDS (PLWHA) have a prevalence of AUDs two to three times that of the general population [3]. Successful HIV disease management can be undermined at each step in the HIV treatment cascade, including linkage, retention, and suboptimal adherence to antiretroviral therapy (ART) [4, 5]. PLWHA with comorbid AUDs tend to fare relatively poorly at each step compared to those without AUDs [6], resulting in a higher likelihood of virologic nonsuppression [4, 7–9]. Though there are conflicting findings from studies using various study designs [9], alcohol consumption is associated with ART medication nonpersistence and nonadherence [10–12]. Drinking [11, 13] or having a treatable AUD [14] is independently associated with ART nonadherence. Thus, while ART has significantly decreased morbidity and mortality from HIV [15], many alcohol-using patients do not achieve the recommended 95% adherence level necessary to maintain viral suppression and the prevention of the development of a resistant virus [16–19].

Alcohol use in PLWHA is also associated with high-risk sexual behaviors [1, 20, 21], increased vaginal viral shedding and heightened risk of HIV transmission [22], infection with other sexually transmitted diseases [23], and “super-/coinfections” (i.e., infection with a new and/or drug-resistant strain of the virus) [24]. In addition, preclinical studies have demonstrated that the deleterious biomedical consequences of chronic alcohol use also impact disease progression by adversely impacting drug absorption and metabolism [25], elevating viral set point [26], and accentuating end-stage wasting [27, 28]. Thus, decreasing AUDs in PLWHA is imperative in order to ameliorate the deleterious biomedical consequences of HIV infection as well as to improve adherence and effectiveness of ART. This will lead to improved quality of life and decreased disease transmission, thus positively impacting the HIV epidemic from multiple angles.

Despite significant evidence of alcohol's negative impact on PLWHA, there is a shortage of interventions targeting PLWHA with AUDs, particularly for use within the context of primary care settings [29–31]. Only a small percentage (5.6%) of HIV-infected in-care patients surveyed by the HIV Cost and Services Utilization Study, a nationally representative sample of PLWHA currently in care, received substance abuse treatment in an outpatient setting, and a mere 12.4% attended self-help groups, primarily due to unavailability of appropriate services [30]. Little is known about PLWHA knowledge regarding the impact of alcohol consumption on HIV medication adherence, treatment outcomes, and disease progression. There is some evidence to suggest that a significant number of PLWHA believe there is a toxic interaction between alcohol and ART, leading to intentional nonadherence on drinking days [32]. A knowledge gap exists in further understanding PLWHA beliefs as well as ways in which to effectively intervene at the level of knowledge/attitudes to reduce the risk of alcohol consumption.

Although not specific to alcohol use, advancements have been made in creating evidence-based health risk reduction interventions that target drug-involved persons [33]. Specifically, the Holistic Health Recovery Program (HHRP+) for HIV-positive drug users is an evidence-based intervention (EBI) program that focuses on harm reduction related to illicit drug use, safer sexual practices, introduction to the 12-step program, negotiation of condom use with partners, managing stress, stigma, and grief, developing healthy social relationships, and engagement in healthcare [33]. This EBI has demonstrated efficacy in reducing injection drug use and improved ART adherence in PLWHA [33, 34]. HHRP+ is a group-based intervention and is grounded in the Information-Motivation-Behavioral Skills (IMB) model, which postulates that a combination of enhanced knowledge, behavioral skills, and motivation is critical in effectively producing actual health behavior change. This program was chosen as research has demonstrated that key IMB model constructs (i.e., motivation, skills, and self-efficacy) play a critical role in the successful modification of HIV-related health behaviors [35], including ART adherence [36], and safe sex behaviors [37].

The purpose of the present study was to conduct the formative research necessary to adapt the HHRP+ to reduce health risks among HIV+ alcohol users participating in treatment in an urban HIV outpatient clinic. We sought to gain information about alcohol-related treatment issues and needs, key characteristics of the HIV transmission profile of the target population, and ways to optimize intervention content, placement, and delivery.

## 2. Methods

### 2.1. Participants

Participants were recruited using flyers posted in a large, urban HIV outpatient clinic in New Orleans, LA. Interested patients contacted the research team who screened them for eligibility, which included the following: (a) aged 18 years or older; (b) being HIV-infected and in care; and (c) consuming at least one alcoholic drink in the past month. Eligible patients (*N* = 37) then underwent informed consent procedures and were sequentially assigned to 7 focus groups (FGs) containing 3-8 participants per group. Similarly, all healthcare providers from a multidisciplinary team at the same clinic were invited to participate; 16 of 45 total providers consented to participate in 3 FGs.

### 2.2. Procedure

All study procedures, including recruitment flyers, were approved by the LSUHSC Institutional Review Board and clinic research committee. The ADAPT-ITT framework was followed for the initial intervention adaptation process [38], as reported here. The ADAPT-ITT model, specifically designed to address the scarcity of HIV-related EBIs, consists of eight phases including* Assessment, Decision, Administration, Production, Topical Experts, Integration, *and* Training*. The* Assessment* phase involved conducting FGs with members of the target population and key stakeholders (i.e., treatment care providers) to determine the specific needs of the HIV outpatient clinic population. In the* Decision* phase, results from FGs were used to inform the selection of the most appropriate EBI to adapt. The* Administration* and* Production* phases involved distilling content from the original EBI as well as creating the first draft of this adaptation. Topical expert's feedback was integrated during the refinement of the adapted EBI. We are reporting the results from the initial four phases involved in adaptation of the intervention.

FGs with patients and healthcare providers were conducted to gain information regarding alcohol-related treatment issues and needs, key characteristics of the HIV transmission profile of the target population, and ways to optimize intervention content, placement, and delivery. FGs were conducted by a doctoral-level clinical psychologist using scripted FG interview guides (Tables 1 and 2). Primary themes derived from focus groups were used to select and modify the most relevant HHRP+ content for use in a pilot test of the adapted intervention.

### 2.3. Data Analysis

FGs were audio-recorded with participant permission and transcribed verbatim. Transcripts were entered into qualitative analysis software, NVivo 10 (QSR International, Burlington, MA), for content analysis. Data for the target population and healthcare provider FGs were analyzed separately. Analyses involved deriving “nodes” or groups of thematically similar content directly from the transcripts. The creation of nodes allowed coders to collect, quantify, and organize all data relevant to that particular theme. The coding process was completed for each individual FG transcript and a master list of themes was compiled to reflect overarching themes. Qualitative coding was conducted by two independent coders and interrater agreement was calculated by a third member of the research team to ensure 95% agreement or greater for each FG. Disagreement amongst coders was resolved through consensus conferences among all three trained members of the qualitative analysis team. FG transcripts were analyzed using content analysis to obtain salient themes from the participants' responses [39, 40]. The transcripts were reviewed using an in-depth, systematic strategy to identify patterns in responses and fit responses into the coding categories while revising preliminary categories to better fit the responses [41, 42]. Responses were organized under broader themes associated with the main topics from the FG protocols. Themes considered predominant or most salient were those that emerged most frequently within and across participant FGs.

## 3. Results

### 3.1. Participant Demographics

Patient participants were primarily male (65%), African American (86%), and in their late 40s (*M* = 48.9 years, SD = 9.2). Patient participant demographics are well representative of the HIV outpatient clinic population. Three healthcare treatment provider FGs were conducted and grouped as follows: primary care (*n* = 7), social services (*n* = 5), and behavioral health and patient education (*n* = 4). The following specialties personnel were represented: infectious disease physician (*n* = 5), nurse practitioner (*n* = 1), nurse supervisor (*n* = 1), social services (*n* = 4), medication coordinator (*n* = 1), clinical psychology (*n* = 2), patient education (*n* = 1), and nutrition (*n* = 1). Provider participants were primarily female (69%) and Caucasian (81%).

### 3.2. Focus Group Interviews with Target Population

#### 3.2.1. Substance Use

Participants were asked the average number of drinking days and days using recreational drugs in the prior month. Regarding alcohol use, responses ranged from 0 to 30 drinking days in the prior month, with the median response being 8 drinking days. On average, participants reported 10.7 (SD = 8.9) drinking days in the prior month and recreational drug use was reported by 35% of participants. Those who endorsed drug use reported engaging in use an average of 13.6 days (SD = 13.2) in the prior month, with marijuana the most commonly used drug (87.5%).

#### 3.2.2. Most Important Problem Faced

When asked, participants indicated that the most important problems they faced related to HIV were stability (e.g., financial and housing) and achieving or maintaining sobriety. A newly diagnosed patient discussed an increase in alcohol use following his diagnosis,

*“The main problem in my life is finding peace with my diagnosis at this point…. I went into self-destruct mode [with alcohol] and sort of like derailed my entire life.”*

Approximately one fourth of participants discussed that their desire for, and pursuits of, stability were more important than issues related to HIV disease management. Stability concerns involved finding permanent housing, financial stability, and locating work appropriate for their current health status. Regarding stability, consider the following:

*“Wanting to work and having my doctors tell me they don't think I should. And then I'm used to working and making money and now I get a check, because I had a stroke, and it's not coming together. I wish I could go back to work and all that. It's coming along but it's just moving slow.” *

#### 3.2.3. Most Common Contributor to Alcohol Use

When asked to describe common moods that precipitated engagement in alcohol use, some participants reported that it was during joyous or celebratory occasions, but most (about 2/3) indicated that their alcohol use was driven by negative mood states, including stress and depression. For some, drinking behavior worsened in response to news of HIV diagnosis:

*“Um, well, I've kind of been in a war zone since I found out that I was positive and so drinking like every time I drink now, like, I'm aware that it's for a specific reason and it's to kind of soothe whatever psychological and emotional impasse that I feel I'm at. Like when I found out, like, I spent two weeks solid like drunk and that's how I lost my job. And so, I, you know, I like drink now for, um, purely kind of like therapeutic…”*

Others reported that alcohol use served as an escape from feelings of grief:

*“When I think about my mama, because it's going to be going on a year, so that make me – that make me drink a – drink a lot, because I think about her, because I miss her, you know.” *

#### 3.2.4. Discussions about Alcohol Use with Providers

Most participants (65%) specifically acknowledged that, at some point in the course of their healthcare, they engaged in discussions with their provider about their alcohol use. Those who frequently discussed alcohol intake commonly had either a substance abuse history and/or other comorbidities in addition to HIV/AIDS:

*“As for me, I've discussed it a lot recently, because I've also been diagnosed with hepatitis, and they were saying that the alcohol would really affect my liver.” *

Greater than 20% of participants also shared that while their alcohol use may have been assessed by their providers, the subsequent discussion was minimal:

*“My doctors, they don't discuss it with me. They just ask me questions like, do I drink, and I say yes or no. And then they just leave it alone.”*

Despite their interest in methods for reducing alcohol use, some participants felt that they did not receive any guidance regarding ways in which they could attempt to decrease their intake.

*“Well, they tell me I need to stop, but not – no – no ideas, no nothing.”*

#### 3.2.5. Knowledge regarding Interaction between Alcohol and Antiretroviral Medications

We sought to understand the level of knowledge currently held by participants in order to identify knowledge gaps to be targeted in the adapted intervention. Participants were asked to describe their knowledge regarding ways in which alcohol interacts with antiretroviral medications and the responses varied significantly. Many participants (41%) were not aware of any negative impact of alcohol on ART:

*“I read the pamphlets that come with it, the medication that we do, and the three meds that I'm on, it doesn't say anything about drinking alcohol, and it doesn't say anything with the effect it's going to have on you if you drink alcohol.” *

Others specifically denied this relationship:

*“Basically I don't think being an alcoholic really messes it up. It's the crack [cocaine] and stuff –”*

Of those who did acknowledge a detrimental impact of alcohol use, the most common response (greater than 1/3 of participants) was that drinking can negatively affect or “weaken” medication effectiveness; however, participants often continued to engage in alcohol use unless they experienced negative physical consequences such as lightheadedness, nausea, or vomiting. Several participants who have experienced these negative physical reactions when mixing antiretroviral therapy (ART) and alcohol reported that they have skipped doses of their medication when they were intoxicated, planned to drink, or were recovering from the effects of drinking. One participant stated:

*“The alcohol intensifies the side effects – the dizziness, the nightmares, the restlessness. That's why if I really plan on drinking a lot I don't take it [ART]. I don't like that feeling.”*

Two participants, however, stated that they believed alcohol was beneficial to ART:

*“The medication, once you take the medication, it absorbs quicker in your system when you drink with it and it starts going straight to your bloodstream. That's what it does to me. When I take mine with beer or something, it goes straight to my bloodstream and it absorbs quicker because they have acid in their beer and you got acid in your body ‘cause they have – it's acid and it breaks down the medication quicker.”*

#### 3.2.6. Knowledge regarding the Impact of Alcohol on HIV Disease Progression

Participants were also asked to discuss their knowledge related to alcohol's impact on HIV disease progression. Although many participants reported that they had never been informed of any HIV disease-related consequences of alcohol use, the general consensus among those who had some basic knowledge regarding this concept was that alcohol “speeds up” HIV disease progression:

*“Because it's no good for you. You're already fighting illness inside of you, diseases and stuff. HIV itself is eating away cells in your cells you going on with alcohol or drugs, that ain't going to make it no better. It makes it worse. I see people too die quicker on drugs and alcohol. You know it. They die quicker while on – especially alcohol. Alcohol is worse than drugs when you're HIV.”*

Others made a direct connection between alcohol use, decreased medication effectiveness, and disease progression:

*“Yeah, I believe it will, because – because if you're drinking alcohol, and if you're trying to take your medicine, the alcohol going – going to blunt the effect of the medicine.” *

#### 3.2.7. Participant Desire for Alcohol and HIV-Related Education

During the discussion regarding alcohol's impact on HIV disease progression and ART, participants were asked to comment on whether or not they would be interested in learning more about these concepts. All participants unanimously responded that they would like more alcohol and HIV-related knowledge. Several participants noted that they planned to pursue this information in the near future:

*“A lot of people that do have that [HIV/AIDS], they don't know this. It's a good thing to know. I'm going to ask my primary care physician and my HIV physician.”*

#### 3.2.8. PLWHA Commentary on Intervention Advertisement and Delivery

Last, patient participants indicated that they would prefer a harm reduction intervention as opposed to an intervention that focuses on alcohol abstinence. Patient participants also provided suggestions regarding ways to optimize the acceptability of study advertisement, preferring a holistic intervention that focused on a variety of health-related topics. They also described that advertisement for the study should state that the goal of the intervention is to improve overall health and well-being as opposed to advertising it as a substance-use or HIV program.

### 3.3. Focus Group Interviews with Treatment Providers

#### 3.3.1. Reasons for Patient Engagement in Risky Behavior

The majority of providers who offered insight into this topic believed that their HIV+ patients engaged in risky substance use and unsafe sexual behavior due to habit and that these were the same behaviors that led to HIV infection. Responses included the following:

*“They've already kind of created a pattern of behavior that probably is what caused them to get the virus in the first place.”*

*“Yeah I agree with the habit part too, because you know like they were practicing risky behaviors for so long beforehand and then as far as they're concerned, the worst thing that could have happened, happened to them already.”*

Others believed that risky behaviors were more common in younger patients,

*“I think my younger group, the younger population of our HIV… It's basically, I'm not going to be stigmatized or labeled that I am HIV positive so I'm just gonna act like everybody else is acting.”*

#### 3.3.2. Provider Insight regarding PLWHA Alcohol Use

Additionally, providers discussed possible determinants specifically for heavy alcohol use in their patients. They noted that common stressors such as poverty, chronic unemployment, and boredom may contribute to the use of alcohol as an inexpensive coping mechanism.

*“I would say it's the same reason anybody becomes alcoholic. I mean, HIV, HIV positive and negative can become alcoholic for the same reason this genetic tendency to become addicted earlier or easier and then the reasons I..I think it's mostly to escape.” *

They also noted that their patients often rely on alcohol as a coping mechanism and that this may exacerbate feelings of hopelessness and resistance to change drinking habits. As a result, all providers agreed that the adapted intervention should involve development of stress management skills.

#### 3.3.3. Permissiveness and Pervasiveness of Drinking

All providers agreed that the local (New Orleans, LA) drinking culture is a contributor to heavy alcohol use in their patients living with HIV/AIDS. For example, many providers mentioned that beer and wine are often not considered alcoholic beverages and that this belief is not exclusive to their patients. Typical responses included the following:

*“You gotta realize that, they say that wine is not an alcohol. I mean, in their minds they don't consider it. But in the minds of the professionals that work here too, it's not an alcohol!”*

Others commented on cultural norms related to drinking,

*“We have a lot of festivities where…^*∗*^*laughterfromgroup*^*∗*^ like Mardi Gras where the norm is to drink. The culture of the area sets it up to do a lot of parties.” *

Some providers gave specific examples of these alcohol-related practices,

* “Growing up in a culture like this, even the kids' parties that they have (alcohol). You go to a kid's or a toddler's party and you know they have the stuff for kids and they got the stuff for adults too! Which it always includes alcohol, so it's like, from birth, that's the way it is.” *

#### 3.3.4. Barriers to Patient-Provider Alcohol Discussions

Providers were asked to comment on the level of difficulty engaging in conversations about alcohol use with their patients. Several indicated that simply asking about alcohol use is not difficult, but that the challenge is in gaining accurate information from their patients:

*“If you want an honest answer, you might find it difficult. But if you just say ‘how much you drinking? Oh not much.' Okay.”*

Providers also stated they may not discuss alcohol use in great detail with their patients for fear of disrupting delicate relationships.

*“I have to be honest—I feel more comfortable with patients that are relatively new to me than patients that I've been seeing for a long time and I'm worried that they're starting to drink too much. It kind of makes me uncomfortable to talk to them because I'm worried that it's going to disrupt the long-term relationship that we have. And I don't normally push it unless there's cause. Like if I'm seeing viral loads that are showing a lot of noncompliance.”*

#### 3.3.5. Provider Difficulty with AUD Diagnosis, Treatment, and Referral

Healthcare providers discussed that they have encountered difficulty in diagnosing AUDs in their patients. This was initially evident as providers in all focus groups were unaware of the diagnostic criteria for AUD. Others noted that because many of their patients are unemployed and/or sedentary, it has been difficult to define functional impairment.

*“They're not working so it's not like their alcohol consumption is keeping them from going to work.”*

Many expressed that they did not feel they had an accurate way to determine the frequency and severity of patient alcohol use,

*“But, I have no idea if drinking is affecting their life. I just, I just don't think I have any way of accurately assessing how much somebody drinks.”*

Most providers indicated that even if they were able to properly identify patients with AUD, they did not feel confident in their ability to effectively provide treatment.

*“I don't know if we have had an impact on folks who, who are using or abusing.”*

Many noted the lack of access to resources as well,

*“We don't have a place to send patients even if they wanted to get clean.”*

### 3.4. Specific Intervention Considerations

Both target population and healthcare provider groups were asked questions regarding intervention length and individual session length, as well as ways to optimize recruitment and intervention participation. Providers were asked to comment on the best days and times to host intervention groups that would maintain a balance between optimal PLWHA participation and avoidance of disrupting particularly busy clinic hours.

Patient participants and providers generally agreed that the adapted intervention should include no more than 5 group sessions ranging from 45 to 90 minutes each. Many PLWHA suggested, however, that they would be willing to engage in a more lengthy intervention. The majority of patients indicated that while few had attended an HIV-related education program in the past, they felt comfortable doing so in a group setting. In addition, feedback was provided regarding intervention delivery such that relying on written materials was discouraged due to low literacy rates and brief, straightforward videos were encouraged to supplement material discussed.

## 4. Intervention Adaptation

We used the ADAPT-ITT framework [38] to adapt the original HHRP+ intervention, an evidence-based risk reduction and treatment adherence intervention, so that it could be implemented to reduce AUDs and reduce health risks among PLWHA in our clinic [33]. Results from the focus groups conducted with patients who drink alcohol and with treatment providers at an outpatient HIV clinic in New Orleans, LA, identified the following key themes regarding our population of PLWHA for inclusion in the adapted EBI: (1) negative mood states are a significant predictor of heavy alcohol use; (2) high levels of psychosocial stress, paired with few adaptive coping strategies, perpetuate the use of harmful alcohol self-medication; (3) local cultural norms related to the permissiveness and pervasiveness of drinking contribute to heavy alcohol use; (4) healthcare providers unanimously stated that outpatient options for AUD intervention are scarce; (5) misperceptions about the relationships between alcohol and HIV are common; (6) patients are interested in learning about alcohol's impact on ART and HIV disease progression; (7) healthcare providers lack adequate training in the assessment of AUDs and this may be ameliorated by helping patients identify when their alcohol use is problematic.

Focus group content was used to determine how to best adapt the original HHRP+ content. Based on feedback from these FGs, the pilot version of our HHRP+ AUD intervention, the WELL Program, consists of five group sessions, each lasting approximately 90 minutes. The WELL Program focuses on issues pertaining to alcohol use, ART adherence, relapse prevention, and HIV-risk reduction and integrates science-derived information related to alcohol and HIV biomedical consequences obtained from the nonhuman primate model of chronic alcohol and disease progression [25–27]. The intervention consists of the following adapted sessions (Table 3): (1) healthcare participation; (2) reducing the harm of alcohol and drug use; (3) harm reduction with latex; (4) negotiating harm reduction with partners, and (5) healthy lifestyle choices.

## 5. Discussion

This study illustrates the process of conducting the formative research needed to properly adapt an EBI for PLWHA who may have problems surrounding alcohol misuse. Results from our focus groups suggest that addressing alcohol use in PLWHA should involve a brief and targeted harm reduction intervention that can be delivered in a primary care setting with optimal accessibility to the target population. Because HHRP+ has demonstrated effectiveness in reducing injection drug use, improving medication adherence, and decreasing risk behaviors in PLWHA [33], it serves as an appropriate base for integrating results from our formative FGs.

Our results suggest that most PLWHA in our clinic are unaware of the specific consequences of heavy drinking, particularly as it relates to the impact on ART effectiveness and HIV disease progression, as is consistent with prior studies [43]. Our findings are also in line with prior research regarding the impact of PLWHA alcohol and ART toxicity beliefs on ART adherence [32] and provide further support for intervention content targeting these information deficits. Our results suggest that these information deficits may be due to healthcare provider lack of discussion regarding alcohol use if (a) the patient is not currently intoxicated or does not have an overt alcohol problem or (b) the provider fears the possibility of disrupting the patient-provider rapport.

Our findings further elucidate the role of cultural norms in contributing to heavy drinking by PLWHA in our clinic. For example, it was surprising to learn that some patients do not believe wine is alcohol; therefore, our intervention content will address this by providing education regarding the types of alcohol, strength of alcoholic beverages, and standard serving sizes so that PLWHA can accurately assess their alcohol consumption. Our findings also highlight the role of negative mood states in contributing to heavy alcohol use, especially when paired with poor coping abilities. Therefore, in addition to maintaining key components of HIV risk reduction interventions (e.g., healthcare participation, harm reduction with latex, and negotiating condom use), it was determined through our focus groups that it is critical to include skill building in the area of stress management/coping as well as targeted information regarding the specific biomedical consequences of heavy alcohol use in PLWHA.

Findings from our FGs also highlight a disconnect in what patients and providers report with regard to interventions focusing on alcohol consumption. Our patients living with HIV/AIDS commonly reported that discussions regarding their alcohol use were limited, and if it was identified that their drinking may be harmful, that they rarely received practical guidance for how to reduce their drinking. On the other hand, providers reported that they felt unprepared to both assess and refer for AUDs and that they perceived that their patients may be unreceptive to strategies or information about alcohol reduction. This discrepancy will be addressed both through patient training in effective communication skills with healthcare providers in the WELL Program and through provider-focused interventions that are currently being developed for future use. Provider-focused interventions will also address optimal timing for patient intervention as well as increasing the confidence of providers in discussing alcohol use with PLWHA as it was noted in our FGs that providers experience a level of discomfort with whom they are familiar.

Although we were successful in gathering data pertinent to the information, motivation, and behavioral skills needs of this population, we acknowledge some limitations to our findings. First, it is possible that there may be self-selection bias present in the patients who participated in the focus groups. These may represent a subset of individuals within the target population who express greater motivation and healthcare participation as they volunteered to attend and respond to focus group questions. Despite this possibility, we made efforts to ensure that all qualifying patients had an equal opportunity to participate through the use of flyers, monetary incentives, and flexibility in scheduling of focus group meetings, and participation was independent of literacy level. There may also have been response bias inherent in the FG questions asked and the setting in which the FGs were held. For example, although confidentiality was emphasized in our FGs, it is possible that participants were hesitant to fully disclose sensitive information in the context of the group setting and in the clinic building itself. Relatedly, we did not gain information regarding the stigmatization of PLWHA who drink alcohol nor did we comprehensively assess their patterns of alcohol consumption. We believe these topics would be useful to explore in future studies. We also acknowledge that we may have limited generalizability to populations such as those with different racial or ethnic backgrounds, as the majority of our target population participants were African American. We failed to discuss preferences for gender-specific or mixed-gender intervention sessions in our focus groups and believe this is a limitation as well. Additionally, we have yet to determine whether our resulting intervention will translate into significant health-related changes in our patient population. Consistent with the ADAPT-ITT framework [38], however, this is currently being examined in a pilot study to test the feasibility, acceptability, and patient satisfaction related to the adapted intervention. Following the pilot phase, participant feedback will guide intervention modifications in preparation for a randomized controlled trial (RCT). The RCT will determine the efficacy of the adapted HHRP+ AUD intervention as compared to treatment as usual in increasing the proportion of PLWHA achieving viral suppression, optimizing ART adherence, and decreasing alcohol use by modifying information, motivation, and behavioral factors associated with HIV transmission and progression.

Together, findings from our formative research highlight the need for EBIs focusing on decreasing AUD in PLWHA and provide vital information regarding intervention content, placement, and delivery. Building on existing EBIs in order to optimally address population-specific risk behaviors such as AUDs among PLWHA is critical in order to prevent or ameliorate the deleterious comorbid consequences of HIV infection to PLWHA, to improve their ART adherence, and to ultimately improve community health.

## Figures and Tables

**Table 1:** Focus group script for target population.

General information	(1) How much have you discussed issues about alcohol use and HIV with your treatment providers in the past?
	(2) What is the most important problem in your life right now?
	(3) What do you value most?

Alcohol history	(4) In the past month, about how many days did you drink alcohol?
	(5) Did you drink to get drunk? Is this normal for you or does this happen often?
	(6) When do you find yourself drinking the most? What moods, times of day, situations, or with what people?
	(7) Do you think drinking gets in the way of you taking care of yourself?
	(8) Has drinking ever interfered with your life responsibilities or gotten you into any legal trouble? Have you ever thought your drinking was a problem?
	(9) Have you ever tried to cut back on or stop your drinking? If so, how? Did it work? What got in the way?
	(10) Do you think your drinking experience is like other people in your life?

Alcohol and HIV-related knowledge	(11) Do you know how alcohol interacts with your medications?
	(12) Do you know how alcohol affects the progression of your illness?
	(13) Would these questions (11 and 12) be good to address specifically during our intervention program?
	(14) Have your health problems motivated you to cut back on or quit your drinking?
	(15) Did you know that being HIV+ puts you at higher risk for poor health when you drink or use drugs and is unsafe about sex? Why do you think most people still do it?

Substance abuse history (excluding alcohol)	(16) In the past month, how many days did you use any recreational drugs, including any pills that were not prescribed to you? If any, what kind? Is this normal for you?
	(17) Has using drugs ever gotten in the way of keeping up with your life responsibilities? Have you ever thought it was a problem? (a) If yes, have you ever tried to cut back or stop your drug use? If so, how? How did it work? What helped/what got in the way? (b) If yes, do you seem to drink more when you are using drugs? Or vice versa?
	(18) Do you think recreational drug use is common for your friends/peers/others in the clinic? How much?

Intervention-specific	(19) Have you ever participated in an HIV education program that covered alcohol and drug use too? Was it helpful? What did you like best or what did not you like?
	(20) What helps you remember things best? Seeing or hearing information? Participating in activities?
	(21) Do you feel OK in a group setting?
	(22) How long should each session last?
	(23) How many sessions should our program be?
	(24) What time of the day should the sessions be?
	(25) What would be a good way to make sure participants attend these sessions?
	(26) What would get in the way of you coming to these sessions?

**Table 2:** Focus group script for treatment care providers.

Patient HIV-specific knowledge and risk behaviors	(1) What do you think is your patients' level of understanding about HIV transmission risk?
	(2) What types of HIV risk behaviors do you perceive in your patients?
	(3) Why do you think patients practice risky behaviors? What kinds of situations seem the most common?
	(4) What do you think is the role of information deficits in leading to risky behavior? What specific information deficits do you think your patients have?
	(5) What do you think is the role of motivational obstacles in contributing to risky behavior?
	(6) What do you think is the role of behavioral skills deficits in contributing to risky behavior? What specific behavioral skills deficits do you think are present?

Perception of patient alcohol use	(7) Approximately how many patients in the clinic do you think are diagnosed with alcohol use disorders?
	(8) What do you think contributes most to excessive drinking in your patients?

Perception of patient alcohol and HIV-related knowledge/attitudes	(9) Do you think there are any negative attitudes towards safer sexual and substance use behaviors that patients possess? If yes, what are they?
	(10) Do you think there are any norms that patients have that interfere with safer sexual and substance using behaviors? If yes, what are they?

Current practice in the clinic	(11) Do you regularly discuss alcohol use with your patients? If so, how often?
	(12) Is discussing alcohol use difficult to navigate as a provider?
	(13) What HIV prevention approaches do you use right now? How well do they work? Are there any improvements that could be made that would make them more effective?
	(14) What has worked best to decrease substance abuse (particularly alcohol) or to elicit other behavioral changes like HIV risk reduction with your patients? What has been tried that was not successful?

Intervention-specific	(15) What type of intervention would work best? Individual or group?
	(16) How long should each session last and how many intervention sessions seem reasonable?
	(17) What time of the day should the sessions be in order to not interfere with clinic operations?
	(18) What day of the week would work best to gain patient participation?
	(19) What mode of presentation would work best?
	(20) How would this intervention be best implemented in this facility without disrupting the routine?
	(21) What would be a good way to make sure that patients attend these sessions?

**Table 3:** Overview of adapted intervention sessions.

Session topic	Objectives
(1) Healthcare participation	(i) Learn to be an active healthcare participant (ii) Learn the consequences of ART nonadherence and strategies for overcoming barriers to adherence

(2) Reducing the harm of alcohol and drug use	(i) Learn standard drink amounts and identify problematic alcohol use (ii) Understand the harms of alcohol and drug use (iii) Learn alcohol harm reduction techniques

(3) Harm reduction using latex	(i) Identify the harm of unsafe sexual practices (ii) Learn harm reduction techniques using latex

(4) Negotiating harm reduction with partners	Improve condom negotiation and communication skills using didactic, visual, and role-play demonstrations

(5) Healthy lifestyle choices	(i) Learn coping skills and stress management (ii) Improve knowledge regarding nutritional guidelines and food hygiene
